# Maternal smoking in pregnancy association with childhood adiposity and blood pressure

**DOI:** 10.1111/ijpo.12046

**Published:** 2015-07-14

**Authors:** L. Li, H. Peters, A. Gama, M. I. M. Carvalhal, H. G. M. Nogueira, V. Rosado‐Marques, C. Padez

**Affiliations:** ^1^ Population, Policy, and Practice ProgrammeInstitute of Child HealthUniversity College LondonLondonUK; ^2^ Departamento de Biologia AnimalFaculdade de CiênciasUniversity of LisbonLisbonPortugal; ^3^ Research Centre for Anthropology and HealthDepartment of Life SciencesUniversity of CoimbraCoimbraPortugal; ^4^ University of Tras‐os‐Montes and Alto DouroVila RealPortugal; ^5^ Department of GeographyUniversity of CoimbraCoimbraPortugal; ^6^ Tropical Research Institute of LisbonLisbonPortugal

**Keywords:** Adiposity, blood pressure, children, maternal smoking in pregnancy

## Abstract

**Background:**

Maternal smoking during pregnancy has been associated with increased risk of childhood overweight/obesity defined by body mass index (BMI). We examined its association with a range of adiposity measures and cardiovascular indicators in children aged 3–10 years.

**Methods:**

We used data from a cross‐sectional study of schoolchildren across mainland Portuguese districts (2009–2010). We applied quantile regressions to examine maternal smoking associations with adiposity (*n* = 17 286), blood pressure (BP) and resting pulse rate (RPR) (*n* ≈ 2500) measures across the age range, adjusting for prenatal and early life factors.

**Results:**

Maternal smoking during pregnancy was associated with increases in offspring adiposity levels. The difference in median BMI between children of smokers and non‐smokers was 0.39 kg m^−2^ (95% confidence interval: 0.25, 0.53) in boys and 0.46 kg m^−2^ (0.31, 0.62) in girls; 0.55 cm (0.24, 0.87) and 0.82 cm (0.45, 1.19), respectively, in median waist circumference; and 0.94 mm (0.49, 1.40) and 1.47 mm (0.87, 2.07) in median sum of (triceps, subscapular, suprailiac) skin‐folds. The associations appeared to be stronger with increasing age. The differences in the 90th centile tended to be greater than those in median. There was no consistent association of maternal smoking with BP and RPR.

**Conclusions:**

Children whose mother smoked during pregnancy had higher adiposity levels than children of non‐smokers, across several measures, particularly among older children. Although there was no consistent association with cardiovascular indicators, maternal smoking association with childhood obesity may have implications for cardiovascular risk factors over the life course.

## Introduction

There is increasing evidence to suggest that maternal smoking during pregnancy is associated with increased adiposity levels and risk of overweight/obesity in offspring. The associations persist after adjustment for birth size and socioeconomic factors 1, 2. Research into maternal smoking and offspring adiposity tends to focus mainly on body mass index (BMI) as an indicator of total adiposity at one age in childhood 2. Less is known about whether the maternal smoking/adiposity association differs by age. A recent study found little or no association with BMI in infancy 3, while others show that greater BMI and risk of overweight are evident by 2–3 years 4, 5 and perpetuate as the child ages 5. A longitudinal cohort study shows the association is stronger with offspring BMI in adulthood than in childhood 6.

Limited evidence is available for the association of maternal smoking in pregnancy with other adiposity measures and findings are less consistent. One study shows no association with childhood overweight/obesity defined by skin‐folds 7, whereas another study found an association with increased sum of triceps and subscapular skin‐folds, but no relationship with central adiposity 4. It has been suggested that skin‐fold thickness is more strongly associated with body fatness than is BMI, as estimated by various reference methods 8. Because of these stronger associations with body fatness, it is frequently assumed that skin‐fold thickness would be a better predictor of adverse health outcomes than BMI. Waist circumference, a measure for central adiposity, has been found to be a better predictor of cardiovascular risk factors in children than BMI and the association with high‐density lipoprotein cholesterol and systolic blood pressure (SBP) is independent of BMI 9.

Maternal smoking in pregnancy has been associated with adverse cardiovascular risk factors in offspring, although evidence is scant and inconsistent. Studies reported an increase in mean SBP by ≈1 mmHg in 5‐ to 6‐year‐old children whose mothers smoked in pregnancy compared with those of non‐smokers 10, whereas others found no association 11.

The associations of maternal smoking with childhood adiposity and cardiovascular indicators are of particular interest in the light of substantial increases in child obesity. We aimed to examine (i) whether maternal smoking in pregnancy was associated with adiposity measures (BMI, waist circumference, skin‐fold thickness) and cardiovascular indicators (BP, resting pulse rate [RPR]) in children and (ii) whether the associations differed by the age of the child.

## Methods

We used data from the Portuguese Prevalence Study of Obesity in childhood, a cross‐sectional study carried out in public and private schools across all mainland Portuguese districts (March 2009 to January 2010). The study population was selected based on proportionate stratified random sampling to provide a national representative survey of 3‐ to 10‐year‐old children. In each district, schools were randomly selected from the central database of schools of the Ministry of Education until the established number of subjects was reached. A total of 17 509 children were included. Parents were asked to complete a questionnaire about family characteristics including sedentary and physical activity behaviours of the child. We restricted our analysis to children between 3 and 10 years (mean 7.0 years, <1% under 3 years or over 10 years). The study protocol was approved by Direcção Geral de Inovação e Desenvolvimento Curricular. Informed consent was obtained from all parents.

### Adiposity measures

Trained technicians performed anthropometric measurements using standardized procedures, with children lightly dressed without shoes. Height (to nearest millimetre) was measured with a portable stadiometer and weight (0.1 kg) with an electronic portable scale. BMI (kg m^−2^) was calculated for each child. Waist circumference (mm) was measured with a flexible non‐elastic tape. Triceps, subscapular and suprailiac skin‐fold thickness (mm) were all measured twice with a skin‐fold calliper. The average of two readings was used for each skin‐fold thickness. The sum of three skin‐folds was derived.

### Blood pressure and resting pulse rate

During the survey, one district was chosen from one large region in Northern, Central and Southern Portugal in order to select children from different socioeconomic backgrounds. Clinic measurements were obtained for children in the subsample. SBP, diastolic BP (DBP, mmHg) and RPR (bpm) were measured three times by trained technicians using an Omron M7 BP monitor (Omron Healthcare Co., Ltd. Kyoto, Japan), with the child seated after at least 5‐min rest. We used the average of three measurements in the analysis (*n* = 2492).

### Maternal smoking during pregnancy

Maternal smoking was reported in the questionnaire as whether or not the mother smoked during pregnancy and number of cigarettes smoked per day. Mothers were categorized as non‐smokers (less than one cigarette per day) or smokers (one or more cigarettes per day).

### Covariates

Several potential confounders and risk factors for the outcomes were examined, including maternal BMI, prenatal (maternal age at child birth, birthweight, gestational age, parity) and childhood factors (infant feeding, maternal education, maternal employment, lone mother family, TV viewing time); these were obtained from the questionnaire completed by the parent (mostly the mother). Maternal BMI was calculated from reported height and weight. Parity was coded as first, second and third/higher birth order. Infant feeding was classified as ‘never’ or ‘ever’ breastfed. Maternal education was classified as ‘primary’, ‘secondary’ and ‘university/higher’, and where missing was supplemented with paternal education (1.1%). Parents reported the average number of hours that the child spent watching TV on a weekday, a Saturday and a Sunday. TV viewing time was classified as ‘<1’, ‘1–2’ or ‘≥2’ h day^−1^.

### Statistical analysis

BMI, waist circumference and skin‐fold thickness had a skewed distribution. We thus applied quantile regression models to each outcome (adiposity, BP, RPR measure), separately for boys and girls. To capture the non‐linear trends of these measures for the age range (3–10 years), we adopted a second‐degree fractional polynomial function of age 12. For all the outcome measures, the best fitting fractional polynomials were *a* + *b*·*age* + *c*·ln(*age*) for boys and *a* + *b*·*age* + *c*·*age* ^2^ for girls, based on the residual mean square errors and also Akaike information criterion (AIC) and Bayesian information criterion (BIC). The unadjusted models included the gender‐specific age function and maternal smoking. The models for waist circumference and skin‐fold thickness also included height, as it was positively associated with these measures. We estimated the difference in median and in 90th (top 10%) centiles of each measure between children whose mothers smoked in pregnancy and those of non‐smokers. The models were adjusted first for maternal BMI and prenatal factors, and further for early life factors. The adjusted models for BP and RPR measures also included BMI, which might be on the causal pathway between maternal smoking and cardiovascular indicators. For each outcome, the age interaction with maternal smoking was tested to assess whether the association differed with increasing age.

To illustrate the relative scale of the association between maternal smoking in pregnancy and each adiposity measure, we estimated differences in median and 90th centile for each measure in terms of standard deviation scores (i.e. internally derived standard deviation score [SDS]). We performed additional analysis by grouping children according to mother's smoking: (i) ‘<10’ and ‘≥10’ cigarettes per day to assess whether there was a dose effect; (ii) during/before pregnancy, only before pregnancy and never smoked; and (iii) by excluding children whose mother smoked only before pregnancy to assess whether it had a similar effect as to smoking during pregnancy on offspring adiposity.

Analysis for adiposity measures was based on participants aged 3–10 years with adiposity measures (*n* = 17 287): 16 671 had data on maternal smoking and 11 478 also had complete data on covariates. Analysis for cardiovascular indicators was based on 2492 children with information on BP and RPR measures (*n* = 1832 with complete data). We applied multiple imputation to impute missing covariates to the sample of 17 287 children with adiposity and 2492 with BP/RPR measures. The imputation model included factors predicting non‐response (i.e. sex, age, maternal/paternal education, family size, parity, shared bedroom or overcrowding, lone parenthood), maternal smoking, adiposity measures, cardiovascular indicators and all covariates in the analysis models. We created 10 imputed datasets assuming missing is at random given observed values of other variables. The MIM command in STATA (Stata Corp, College Station, TX, USA) was used to analyse these datasets using quantile regression. Parameters estimated were combined to obtain overall estimates. Analyses were repeated using children with available data. Conclusions were unaltered and hence we present results based on imputation.

## Results

Mothers who smoked during pregnancy (13.7% of the sample) tended to be younger at delivery, have a lower mean BMI and lower education levels, compared with those who did not. Maternal smoking in pregnancy was also associated with higher birth order, lower mean birthweight, premature births (<32 weeks: 1.4 vs. 0.9%, no difference in mean gestational age), non‐breastfed and more TV time (Table 1).

**Table 1:** Mean (SD) and frequency (%) for maternal and child characteristics in Portuguese children whose mother smoked in pregnancy and those whose mother did not

Characteristics	*n* †	All	Non‐smoker	Smoker	Difference*
		(16 680)	(14 397 86.3%)	(2283, 13.7%)	
Maternal BMI (kg m^−2^)	15 633	24.0 (4.0)	24.1 (3.9)	23.3 (4.0)	<0.001
Maternal age (year)	16 300	29.3 (5.3)	29.4 (5.3)	28.7 (5.8)	<0.001
Birthweight (g)	15 965	3213 (524)	3235 (521)	3072 (519)	<0.001
Gestational age (week)	14 672	38.7 (2.0)	38.7 (2.0)	38.7 (2.2)	0.40
<32 weeks		146 (1.0%)	117 (0.9%)	29 (1.4%)	0.03
32–38 weeks		2540 (17.3%)	2170 (17.1%)	370 (18.5%)	
>38 weeks		11 986 (81.7%)	10 380 (81.9%)	1606 (80.1%)	
Breastfeeding	15 958	13 822 (86.6%)	12 044 (87.5%)	1778 (81.1%)	<0.001
Parity	16 198				
First		9008 (55.6%)	7842 (56.0%)	1166 (53.2%)	<0.001
Second		5679 (35.1%)	4959 (35.4%)	720 (32.8%)	
Third or higher		1511 (9.3%)	1205 (8.6%)	306 (14.0%)	
Maternal education	16 318				<0.001
Primary		3032 (18.6%)	2499 (17.7%)	533 (24.1%)	
Secondary		7661 (46.9%)	6513 (46.2%)	1148 (52.0%)	
University		5625 (34.5%)	5098 (36.1%)	527 (23.9%)	
Maternal employment	16 067	13 516 (84.1%)	11 889 (85.4%)	1627 (75.9%)	<0.001
Lone mother	16 317	2313 (14.2%)	1733 (12.3%)	580 (26.0%)	<0.001
TV viewing time	14 939				<0.001
<1 h day^−1^		3619 (24.2%)	3232 (26.0%)	387 (19.5%)	
1–2 h day^−1^		6470 (43.3%)	5645 (43.6%)	825 (41.5%)	
≥2 h day^−1^		4850 (32.5%)	4073 (31.5%)	777 (39.1%)	

*P < 0.05 for difference by maternal smoking based on *t*‐test for continuous variables and on chi‐squared test for categorical variables.

†Children (3–10 years) with adiposity measures. BMI, body mass index; SD, standard deviation.

Maternal smoking during pregnancy was associated with increased adiposity measures (Table 2). The associations tended to strengthen after adjusting for maternal BMI and prenatal factors, and persist when further including early life factors in the models. The adjusted difference in median BMI was 0.39 kg m^−2^ (95% confidence interval: 0.25, 0.53) in boys and 0.46 kg m^−2^ (0.31, 0.62) in girls. The respective difference in waist circumference was 0.55 cm (0.24, 0.87) and 0.82 cm (0.45, 1.19), and in sum of skin‐fold thickness was 0.94 mm (0.49, 1.40) and 1.47 mm (0.87, 2.07). Maternal smoking was also positively associated with individual measure of skin‐fold.

**Table 2:** Differences in measures for adiposity and cardiovascular indicators between children whose mother smoked in pregnancy and those whose mother did not*

	Boys			Girls		
	Model 1	Model 2	Model 3†	Model 1	Model 2	Model 3†
Difference in median						
BMI (kg m^−2^)	0.19 (0.05, 0.33)	0.39 (0.26, 0.58)	0.39 (0.25, 0.53)	0.22 (0.05, 0.39)	0.47 (0.32, 0.62)	0.46 (0.31, 0.62)
Waist (cm)	0.40 (0.10, 0.70)	0.50 (0.20, 0.81)	0.55 (0.24, 0.87)	0.59 (0.25, 0.93)	0.86 (0.54, 1.19)	0.82 (0.45, 1.19)
Sum skin‐folds (mm)	0.86 (0.44, 1.28)	1.02 (0.57, 1.46)	0.94 (0.49, 1.40)	1.10 (0.49, 1.71)	1.49 (0.88, 2.10)	1.47 (0.87, 2.07)
Triceps	0.30 (0.08, 0.52)	0.36 (0.11, 0.61)	0.34 (0.08, 0.60)	0.26 (−0.03, 0.55)	0.53 (0.24, 0.82)	0.57 (0.27, 0.86)
Subscapular	0.26 (0.14, 0.38)	0.27 (0.15, 0.39)	0.24 (0.13, 0.36)	0.34 (0.16, 0.52)	0.47 (0.29, 0.66)	0.40 (0.22, 0.58)
Suprailiac	0.21 (0.57, 0.36)	0.25 (0.10, 0.40)	0.22 (0.08, 0.37)	0.44 (0.22, 0.66)	0.54 (0.33, 0.75)	0.49 (0.27, 0.71)
SBP (mmHg)	−1.20 (−2.90, 0.50)	−0.99 (−3.00, 1.03)	−1.61 (−3.58, 0.36)	−1.44 (−3.64, 0.77)	−1.39 (−3.64, 0.85)	−1.68 (−4.61, 1.25)
DBP (mmHg)	−1.63 (−3.12, −0.14)	−1.59 (−3.10, 0.08)	−1.65 (−2.98, 0.31)	−0.40 (−2.00, 1.20)	−0.35 (−2.02, 1.31)	−0.26 (−1.88, 1.35)
RPR (bpm)	−0.65 (−3.07, 1.77)	−0.46 (−3.06, 2.14)	−0.55 (−3.05, 1.96)	−0.47 (−2.54, 1.61)	−0.33 (−2.61, 1.96)	−0.42 (−2.68, 1.84)
Difference in 90th centile						
BMI (kg m^−2^)	0.82 (0.46, 1.18)	1.04 (0.64, 1.44)	0.84 (0.43, 1.25)	0.39 (0.02, 0.75)	0.63 (0.28, 0.98)	0.63 (0.27, 1.00)
Waist (cm)	1.56 (0.89, 2.21)	1.55 (0.87, 2.22)	1.59 (0.86, 2.33)	0.73 (−0.05, 0.35)	1.39 (0.71, 2.10)	1.14 (0.36, 1.92)
Sum skin‐folds (mm)	2.94 (1.38, 4.50)	2.43 (1.10, 3.76)	2.43 (0.68, 4.18)	1.85 (0.25, 3.45)	3.01 (1.11, 3.90)	1.33 (−0.33, 3.75)
Triceps	0.64 (0.13, 1.14)	0.63 (0.01, 1.25)	0.57 (−0.05, 1.18)	0.06 (−0.45, 0.55)	0.34 (−0.20, 0.89)	0.35 (−0.17, 0.87)
Subscapular	1.31 (0.73, 1.89)	1.23 (0.58, 1.88)	1.16 (0.50, 1.81)	0.77 (−0.02, 1.56)	1.05 (0.33, 1.77)	0.79 (0.06, 1.52)
Suprailiac	1.13 (0.44, 1.81)	0.97 (0.35, 1.60)	0.84 (0.25, 1.43)	0.30 (−0.36, 0.97)	0.86 (0.18, 1.54)	0.75 (0.02, 1.48)
SBP (mmHg)	0.37 (−3.27, 4.02)	0.03 (−4.00, 4.07)	−0.12 (−4.64, 4.61)	−3.88 (−7.41, −0.33)	−4.54 (−7.97, −1.12)	−3.92 (−7.60, −0.24)
DBP (mmHg)	−2.66 (−6.04, 0.72)	−3.01 (−6.21, 0.18)	−3.40 (−6.87, 0.07)	−0.10 (−3.30, 3.10)	0.39 (−3.01, 3.80)	0.65 (−2.57, 3.86)
RPR (bpm)	−0.56 (−3.30, 2.17)	−0.43 (−3.13, 2.26)	−0.63 (−3.31, 2.06)	−0.62 (−3.15, 1.92)	−0.43 (−3.00, 2.15)	−0.33 (−3.08, 2.43)

*Estimated from quantile regression. Model 1 included age, (*t*) trends (*t* + *t* ^2^ for girls; *t* + ln[*t*] for boys) and height (for waist circumference and skin‐folds). Model 2 added maternal BMI, maternal age, birthweight, gestation and parity. Model 3 further added infant feeding, maternal education, maternal employment, lone mother, TV time and BMI (for BP and RPR). Models were fitted using multiple imputation. Sample size differed by adiposity measure (*n* = 17 286 for BMI; 16 568 for waist circumference; 15 329 for skin‐fold thickness) and for BP and RPR (*n* = 2492).

†*P*‐value for age interaction with maternal smoking: for median 0.06 (boys) and 0.04 (girls) for BMI, 0.02 and 0.06 for waist circumference, 0.24 and 0.03 for sum of skin‐folds; *P*‐values > 0.10 for BP and RPR and 90th centiles of all adiposity measures. BMI, body mass index; BP, blood pressure; DBP, diastolic blood pressure; RPR, resting pulse rate; SBP, systolic blood pressure.

Maternal smoking during pregnancy was associated with the upper end of the distribution for each adiposity measure. The adjusted difference in the 90th centile was 0.84 kg m^−2^ (0.43, 1.25) in boys and 0.63 kg m^−2^ (0.27, 1.00) in girls for BMI; 1.59 cm (0.86,2.33) and 1.14 cm (0.36, 1.92), respectively, for waist circumference; and 2.43 mm (0.68, 4.18) and 1.33 mm (−0.33, 3.75) for sum of skin‐folds (Table 2).

There was a positive interaction between maternal smoking in pregnancy and age of the children for median BMI (*P* = 0.06 for boys, 0.04 for girls), waist circumference (*P* = 0.02 for boys, 0.06 for girls) and sum of skin‐folds in girls (*P* = 0.03), indicating that the maternal smoking association with adiposity was stronger among older than younger children. For example, the adjusted difference in median BMI was 0.83 kg m^−2^ (0.48, 1.42) for boys aged 9–10 years, compared with 0.26 kg m^−2^ (0.02, 0.54) for boys aged 3–5 years, and 0.70 kg m^−2^ (0.27, 1.41) vs. 0.20 kg m^−2^ (0.08, 0.50) for girls. There was no interaction between maternal smoking and age for the 90th centile of each adiposity measure.

There was no evidence that the difference in each adiposity measure was greater for children whose mother smoked ‘≥10’ (vs. those of non‐smokers) than children whose mother smoked ‘<10’ cigarettes per day (supplementary Table S1). Relative to children of non‐smokers, the differences in median BMI and waist circumference (not skin‐folds) were greater for offspring whose mother smoked during pregnancy than those whose mother smoked only before pregnancy (Table S2). Furthermore, findings did not change substantially when excluding children whose mother smoked before (not during pregnancy) (Table S3).

There was no consistent association between maternal smoking in pregnancy and the median or 90th centile of SBP, DBP and RPR (Table 2).

## Discussion

This study of Portuguese children aged 3–10 years shows that maternal smoking during pregnancy was associated with increased adiposity levels after adjusting for prenatal and childhood factors. The difference was 0.4–0.5 kg m^−2^ in median BMI (equivalent to 0.18–0.20 SDS), 0.6–0.8 cm in median waist circumference (0.11–0.16 SDS) and 0.9–1.5 mm in median sum of skin‐folds (0.09–0.11 SDS), and appeared to be greater with increasing age. The difference in the 90th centiles was 0.6–0.8 kg m^−2^ (0.29–0.40 SDS), 1.1–1.6 cm (0.19–0.25 SDS), 1.3–2.4 mm (0.18–0.24 SDS), respectively, greater than those in medians. There was no consistent evidence of an association between maternal smoking and offspring BP or RPR.

The strengths of our study are the large national sample of contemporary Portuguese children with a range of adiposity measures, which, although inter‐related, indicate different aspects of adiposity. Although BMI reflects overall adiposity, waist circumference measures central adiposity and skin‐fold thickness is a proxy for fat mass. Children were between 3 and 10 years, allowing us to compare maternal smoking association with adiposity measures by age. Limitations include the cross‐sectional design; thus, we cannot explore growth trajectories. Maternal smoking during pregnancy was ascertained retrospectively. It has been suggested that retrospective reports of maternal prenatal smoking are acceptable to be used to identify its potential associations with child outcomes 13. In a meta‐analysis, the estimated association with increased adiposity levels in offspring changed a little when excluding studies in which mothers reported prenatal smoking at the same time outcomes were measured 14. The timing of smoking during pregnancy was not available in our study. Only a subsample of children had BP/RPR measures. The subsample remained broadly representative of the population sample; the associations between maternal smoking and adiposity measures found in the subsample were similar to those in the population sample, although some groups were overrepresented, e.g. older mothers (mean age 30.1 vs. 29.3 years) and mothers with university degree (45.2 vs. 34.5%). The smaller sample size would reduce the power to assess the associations. For example, the reduction in the 90th centile of SBP in girls of smokers could be a chance finding due to the small number of girls with BP measures born to smokers.

### Comparison with other studies

We found a positive association of maternal smoking in pregnancy with adiposity levels in offspring. Children of smokers had a higher median BMI by 0.4–0.5 kg m^−2^ than those of non‐smokers. A US study showed a difference in mean BMI of 0.1 kg m^−2^ at 4 years and 0.15–0.3 kg m^−2^ at 8 years 15. Our results indicate that the difference was greater among older than younger children across adiposity measures, although we could not conclude that the maternal smoking/adiposity association strengthened with age based on cross‐sectional data. However, a stronger association with mean BMI with increasing age has been found in US children. Although the explanation is not clear, it could be the result of the accumulation of a small change of metabolism or behaviour that becomes detectable over time 15, or it might reflect different postnatal risk factors at different childhood ages.

In our study, the association was evident with both the central and upper end of the distribution for adiposity measures. An association between maternal smoking and a greater 90th centile of BMI distribution is consistent with studies showing an association with increased risk of overweight/obesity, defined by age‐ and gender‐specific BMI cut‐offs 2, 3, 6, 7, 15, 16, 17. A recent meta‐analysis shows that offspring whose mothers smoked in pregnancy were at elevated risk for overweight at 3–33 years 14.

We found an association of maternal smoking during pregnancy with increased waist circumference and skin‐fold thickness. Sum of skin‐folds (subscapular and triceps at 5 years 1, biceps, triceps, subscapular and suprailiac at 6 years 18) was higher for children of smokers, compared with those of non‐smokers. It has also been shown that children exposed to maternal smoking in pregnancy had higher values of total fat mass and also lean mass at mean age 9.9 years than those not exposed 19. Another study found an association of maternal smoking with increased sum of skin‐folds, but not with central adiposity 4.

We found no consistent association between maternal smoking during pregnancy and BP or RPR. Results from previous studies have been inconsistent. Some showed an increase in BP in children whose mother smoked in pregnancy 10. Intrauterine exposure to nicotine has been found to have a direct effect on the cardiovascular system development in rats 20. However, other studies reported no association between maternal smoking and offspring BP 11, 21. A similar association of parental prenatal smoking with offspring BP found in a study suggests that the association of maternal smoking with BP may not be a biological influence on the intrauterine environment 21. There is little evidence on the association between maternal smoking and RPR. Only one study showed a reduction in RPR in smoke‐exposed infants 22.

### Potential explanation for the maternal smoking/adiposity association

Our data provided little evidence of a dose–response effect. However, there were only a small number of heavy smokers in pregnancy (3% smoked ≥10 cigarettes per day). Maternal smoking is a well‐established risk factor for intrauterine growth retardation, including reduced birthweight 17. The majority of studies have shown a positive relationship between birthweight and later adiposity indices 23. Like several other studies 1, 6, we found that the association between maternal smoking and adiposity levels in children strengthens with adjustment of birthweight. This suggests that the risk of childhood overweight/obesity associated with maternal smoking was independent of intrauterine growth.

The mechanisms for the association are not fully understood. Although offspring of smokers had higher adiposity levels than those of non‐smokers, mothers who smoked during pregnancy had a lower BMI than those who did not. In adults, nicotine is associated with increased energy expenditure 24 and could also reduce appetite and weight short term 25. One possible explanation for the association with high adiposity levels in offspring is that foetal exposure to nicotine may affect the *in utero* development of the hypothalamic function, exerting an impact on appetite control and energy expenditure throughout the life course 26, 27. A recent study suggests that prenatal exposure to maternal cigarette smoking may promote obesity by enhancing dietary preference for fat through neural mechanism involving in the amygdala 28.

The association between maternal smoking and offspring adiposity could also be due to maternal factors such as diet, lifestyle, obesity and socioeconomic status, and infant feeding 1, 16. In our study, BMI and waist circumference of offspring whose mothers smoked before pregnancy only (14.9%) differed a little from those whose mothers never smoked. It is likely that women who continued to smoke in pregnancy may have lower valuation of the future, self‐control and risk aversion, and poor family nutritional environment than those who did not. Maternal BMI may act as a proxy for these maternal influences as well as genetic predisposition. We found the association with adiposity measures remained after adjusting for maternal BMI, prenatal and early life factors, although we cannot rule out residual confounding such as maternal or family health behaviour 29. Maternal smoking has been associated with unhealthy diet (i.e. higher intakes of energy, fat, cholesterol and alcohol) 30 and their children are likely to share a similar diet, and these behavioural factors could be important determinants for childhood overweight. Our results may also indicate that quitting smoking, particularly for the duration of pregnancy in women, may have important implications for public health policy.

## Summary

Maternal smoking during pregnancy was associated with increased adiposity levels in contemporary Portuguese children. The association with the median, and more evidently with upper end of the distribution across several adiposity measures, remained after adjusting for prenatal and early life factors. The findings highlight that tackling childhood obesity should focus not only on policies targeting eating behaviours and physical activity of children but also on early interventions of behaviours of pregnant women to effectively reduce the incidence of child obesity. Although there was no consistent evidence of an association with offspring cardiovascular indicators, the underlying role of maternal smoking in childhood obesity may have implications on cardiovascular risk factors over the life course.

## Conflict of Interest Statement

No conflict of interest was declared.

## Author contributions

LL conceptualized the study, designed the analysis, drafted the initial manuscript and approved the final manuscript as submitted. HP carried out the analyses, drafted sections of the manuscript, reviewed and revised the manuscript and approved the final manuscript as submitted. AG, MIMC, HGMN and VR‐M contributed to the data collection, reviewed and revised the manuscript and approved the final manuscript as submitted. CP designed the data collection instruments, coordinated data collection, reviewed and revised the manuscript and approved the final manuscript as submitted.

## Supporting information


**Table S1.** Differences (95% CI)† in 50th centile (median) of adiposity measures and cardiovascular indicators for children whose mothers smoked 1–9 and ≥10 cigarettes per day in pregnancy, compared with children of non‐smokers.
**Table S2.** Differences (95% CI) in 50th centile (median)† of adiposity measures and cardiovascular indicators between children of mother who smoked during/before pregnancy and only before pregnancy, compared with those whose mother never smoked.
**Table S3.** Differences (95% CI) in 50th centile (median)† of measures for adiposity and cardiovascular indicators by maternal smoking in pregnancy, excluding children whose mother smoked before (not during) pregnancy.
