# Supplementary material for: Maternal smoking in pregnancy association with childhood adiposity and blood pressure
Source: Pediatr Obes. 2015 Jul 14;11(3):202–9. doi: 10.1111/ijpo.12046 (PMC4949567; doi:10.1111/ijpo.12046)
Supplement: Supplementary file 1 — Table S1. Differences (95% CI)† in 50th centile (median) of adiposity measures and cardiovascular indicators for children whose mothers smoked 1–9 and ≥10 cigarettes per day in pregnancy, compared with children of non‐smokers. Table S2. Differences (95% CI) in 50th centile (median)† of adiposity measures and cardiovascular indicators between children of mother who smoked during/before pregnancy and only before pregnancy, compared with those whose mother never smoked. Table S3. Differences (95% CI) in 50th centile (median)† of measures for adiposity and cardiovascular indicators by maternal smoking in pregnancy, excluding children whose mother smoked before (not during) pregnancy. [file IJPO-11-202-s001.doc]

**Supplementary tables:**

**Table S1**: Differences (95% CI)† in 50^th^ centile (median) of adiposity measures and cardiovascular indicators for children whose mothers smoked 1-9 and ≥10 cigarettes/day in pregnancy, compared to children of non-smokers

|  | **Boys** |  | **Girls** |  |
| --- | --- | --- | --- | --- |
|  | 1-9/day (11.0%) | ≥10/day (2.8%) | 1-9/day (10.8%) | ≥10/day (3.1%) |
| BMI(kg/m^2^) | 0.32(0.17, 0.48) | 0.40(0.04, 0.77) | 0.48(0.29,0.67) | 0.35(0.04,0.67) |
| Waist circumference(cm) | 0.30(-0.15, 0.75) | 0.73(-0.18, 1.63) | 0.71(0.23, 1.18) | 0.63(-0.28, 1.55) |
| Sum of skinfolds(mm) | 0.62(0.08, 1.16) | 0.71(-0.49, 1.92) | 1.14(0.40, 1.89) | 0.98(-0.26.2.22) |
| SBP(mmHg) | -1.12(-3.15,0.91) | -1.35(-5.28,2.57) | -1.96(-5.07, 1.14) | 0.09(-6.80,6.98) |
| DBP(mmHg) | -0.93(-2.61,0.75) | -1.94(-4.68,0.79) | -0.90(-2.64, 0.83) | 1.67(-2.57,5.91) |
| RPR(bpm) | -0.72(-2.78,1.35) | -1.10(-4.50,2.30) | -0.73(-2.67,1.21) | -1.47(-5.18,2.24) |

† adjusted for age trends (*t*+*t*^2^ for girls; *t*+ln(*t*) for boys), maternal BMI, maternal age, birthweight, gestation, parity, infant feeding, maternal education, maternal employment, lone mother, TV time, and height (for waist circumference and skinfolds) and BMI (for BP and RPR). Models were fitted using multiple imputation.

**Table S2**: Differences (95% CI) in 50^th^ centile (median)† of adiposity measures and cardiovascular indicators between children of mother who smoked during/before pregnancy, only before pregnancy, compare to those whose mother never smoked

|  | **Boys** | **Girls** |
| --- | --- | --- |
| BMI (kg/m^2^) |  |  |
| Before pregnancy | 0.12(-0.01,0.24) | 0.07(-0.03,0.24) |
| Before/during pregnancy | 0.41(0.27,0.54) | 0.47(0.33,0.62) |
| Waist circumference (cm) |  |  |
| Before pregnancy | 0.09(-0.19,0.37) | 0.01(-0.35,0.36) |
| Before/during pregnancy | 0.57(0.27,0.87) | 0.79(0.42,1.17) |
| Sum of skinfolds (mm) |  |  |
| Before pregnancy | 0.72(0.25,1.20) | 0.90(0.28,1.51) |
| Before/during pregnancy | 1.03(0.53,1.53) | 1.23(0.60,1.87) |
| SBP(mmHg) |  |  |
| Before pregnancy | -0.56(-2.25,1.12) | --0.34(-3.15,2.47) |
| Before/during pregnancy | -1.85(-3.64,-0.06) | -1.81(-4.88,1.25) |
| DBP(mmHg) |  |  |
| Before pregnancy | 0.18(-1.12,1.48) | 0.07(-1.46,1.59) |
| Before/during pregnancy | -1.76(-3.18,0.33) | -0.15(-1.84,1.55) |
| RPR(bpm) |  |  |
| Before pregnancy | -0.02(-1.88,1..83) | -0.08(-1.51,1.35) |
| Before/during pregnancy | -0.75(-2.49,0.99) | 0.73(-2.35,0.88) |

† adjusted for age trends (girls: *t*+*t*^2^ for; boys: *t*+ln(*t*)), maternal BMI, maternal age, birthweight, gestation, parity, infant feeding, maternal education, maternal employment, lone mother, TV time, and height (for waist circumference and skinfolds) and BMI (for BP and RPR). Models were fitted using multiple imputation

**Table S3**: Differences (95% CI) in 50^th^ centile (median)† of measures for adiposity and cardiovascular indicators by maternal smoking in pregnancy, excluding children whose mother smoked before (not during) pregnancy

|  | **Boys** | **Girls** |
| --- | --- | --- |
| BMI (kg/m^2^) | 0.41(0.26,0.55) | 0.48(0.33,0.63) |
| Waist circumference (cm) | 0.62(0.32,0.93) | 0.80(0.42,1.18) |
| Sum of skinfolds (mm) | 0.91(0.42,1.41) | 1.38(0.68,2.09) |
| SBP(mmHg) | -1.89(-3.77,-0.01) | -0.38(-1.89,1.14) |
| DBP(mmHg) | -1.78(-3.16,-0.41) | -0.40(-2.10,1.28) |
| RPR(bpm) | -0.71(-2.53,1.12) | -0.74(-2.37,0.88) |

† adjusted for age trends (girls: *t*+*t*^2^; boys: *t*+ln(*t*)), maternal BMI, maternal age, birthweight, gestation, parity, infant feeding, maternal education, maternal employment, lone mother, TV time, height (for waist circumference and skinfolds) and BMI (for BP and RPR). Models were fitted using multiple imputation
